# Antibacterial Effect of Red Laser Therapy on *Enterococcus faecalis* Using Different Photosensitizers: An In Vitro Study

**DOI:** 10.1155/2022/7408554

**Published:** 2022-02-10

**Authors:** Franz Torres, Adrian Mallma, Americo Munayco, Oscar Sotomayor, Franco Mauricio, Frank Mayta-Tovalino

**Affiliations:** ^1^Academic Department, Faculty of Dentistry, Universidad Nacional Federico Villarreal, Lima, Peru; ^2^Department of Basic Sciences, Faculty of Dentistry, Universidad Nacional Mayor de San Marcos, Lima, Peru; ^3^Vicerrectorado de Investigación, Universidad San Ignacio de Loyola, Lima, Peru

## Abstract

**Objective:**

To assess the antibacterial effect of red laser using different photosensitizers such as methylene blue and malachite green on monoradicular premolars contaminated with *E. faecalis* ATCC 29212.

**Methods:**

This was an in vitro experimental study. Monoradicular premolars (44, 45, 34, and 35) were used, which were treated with ProTaper Next. After instrument change, irrigation, disinfection, and aspiration were performed with 2 ml of 4% NaOCl with a NaviTip 30°G needle (Ultradent, South Jordan, UT, USA). Group 1: RL + MB (red laser associated with methylene blue photosensitizer), group 2: RL + MG (red laser associated with malachite green photosensitizer), and group 3: control (no treatment). The *E. faecalis* strain was cultured on trypticase soy agar (TSB) (Difco, Detroit, MI, USA) and incubated at 37°C for 24 h. After the incubation period, colony-forming units (CFU/ml) of each group were counted using the plate count method. The ANOVA test was used with a significance level of *p* < 0.05.

**Results:**

Group 1 had the lowest antibacterial contamination as it averaged only 530 ± 581.3 CFU/ml, while group 2 had the highest contamination with an average of 1990 ± 542.5 CFU/ml. Comparison revealed that there were statistically significant differences between the RL + MB and RL + MG groups (*p* < 0.001).

**Conclusions:**

Group 1 had the best antimicrobial potential because it presented the lowest contamination in CFU/ml of *E. faecalis* compared to group 2 and group 3.

## 1. Introduction

Disinfection is one of the main purposes of endodontics because the reduction in the number of microorganisms in the root canal system will determine the potential success or failure of endodontic treatment, as in other pathologies of the oral cavity [1–4]. In recent years, a wide variety of techniques and materials have been used to achieve ideal disinfection, with chemical-mechanical preparation being one of the most widely used endodontists [5–7]. However, owing to the resistance of the microorganisms present in root canals, it is often difficult to achieve complete elimination with conventional endodontic techniques [8–10].

*Enterococcus faecalis* has the greatest resistance to endodontic treatment because of its ability to survive in very unfavourable conditions within the root canal system, which is why it has become one of the most prevalent bacterial strains in cases of endodontic reinfection [5–8]. Therefore, the search for new alternative and/or complementary methods to conventional endodontic treatment is essential in reducing the rate of occurrence of endodontic failures. Currently, photodynamic therapy, which can destroy resistant microorganisms by combining the action of a light source with a specific wavelength and a photosensitizer in the presence of oxygen, has been introduced in the field of endodontics [11–14].

Much research has been carried out on different light sources, with low-power diode lasers being the most used in photodynamic therapy. In addition, one of the most studied photosensitizers is methylene blue, which can be activated by a light source at a wavelength ranging from 610 nm to 660 nm (red light) [1–3]. Recently, the antibacterial effect of photodynamic therapy has been studied using lasers with red light wavelengths (660 nm) as light sources, associated with methylene blue as a photosensitizer, against microorganisms present in root canals. These studies have good results owing to the high affinity the two have for each other [7–10].

Another photosensitizer reviewed in the literature is malachite green, which can be activated by different wavelengths. This feature allows malachite green to be more versatile than other photosensitizers in terms of its application, as it can be activated by light sources with different wavelengths, including red light [8–12]. Thus, it is likely that with good absorption to the same red light source, malachite green presents an antibacterial effect like or greater than that of methylene blue.

Therefore, this study aimed to evaluate the antibacterial effect of a red laser using different photosensitizers such as methylene blue and malachite green on monoradicular human premolars contaminated with *E. faecalis* ATCC 29212.

## 2. Methods

### 2.1. Study Design

The study was an experimental, prospective, and comparative study. The experiment was carried out at the microbiology laboratory of *Hospital Nacional Hipólito Unanue*, *Lima*, Peru. The sample size was calculated based on data from a pilot study. The sample size was calculated using the mean comparison formula using Stata v.15, with a minimum size of *n* = 30 monoradicular human premolars divided among the three groups.

  Group 1: RL + MB (red laser associated with methylene blue photosensitizer)
  Group 2: RL + MG (red laser associated with malachite green photosensitizer)
  Group 3: control (no treatment)

#### 2.1.1. Selection Criteria

Inclusion criteria
  - Permanent monoradicular premolars recently extracted for orthodontic reasons
  - Premolars with single and straight canals
  - Premolars without the presence of root fractures
  - Premolars without calcifications
Exclusion criteria
  - Premolars with extensive carious lesions
  - Premolars with curvatures
  - Premolars with previous endodontic treatment
  - Monoradicular premolars with hypercementosis
  - Biradicular premolars
  - Roots with incomplete apical formation
  - Roots with signs of external and/or internal resorption

### 2.2. Specimen Preparation

Recently extracted permanent monoradicular premolars were collected for reasons unrelated to the present study (orthodontics). The teeth were then immersed in a container with a 4% sodium hypochlorite solution for 2 h to eliminate impurities and were preserved in saline at room temperature. Once the samples were selected, the crowns were sectioned using a Marathon handheld micromotor (Saeyang Microtech Co., Ltd., Daegu, South Korea) and a diamond disc (Hm22D20) at 35000 rpm to standardize their length to 15 mm.

### 2.3. Endodontic Treatment

The working length was measured using a #10 type K file (Dentsply/Maillefer, Ballaigues, Switzerland), and mechanical instrumentation was performed up to 1 mm before the apical foramen. The root canals (premolars 44, 45, 34, and 35) were prepared and standardized up to the diameter of file #40 using the Protaper Next rotary system (Dentsply/Maillefer, Ballaigues, Switzerland). At each instrument change, irrigation and aspiration were performed with 2 ml of 4% NaOCl with a NaviTip 30G needle (Ultradent, South Jordan, UT, USA). At the end of the preparation, the canal was irrigated with 3 ml of 17% EDTA (Maquira, Maringá, Brazil), followed by 5 ml of physiological solution and dried with sterile paper cones (Spident Co., Ltd., Incheon, South Korea). During the preparation, EDTA was applied to eliminate the formation of the smear layer and then proceed with the infection with *E. feacalis*. Next, each tooth was placed vertically in previously labelled cryovial tubes and fixed with blocks of a Zetaplus silicone material (Zhermack SpA, Badia Polesine, Italy). They were then placed in an autoclavable box, and the samples were autoclaved at 121°C for 20 min.

### 2.4. Inoculation of *E. faecalis*

*Enterococcus faecalis* (strain ATCC 29212) was obtained from the GenLab laboratory. The strain was grown on trypticase soy agar (TSB) (Difco, Detroit, MI, USA) and incubated at 37°C for 24 h. Furthermore, the bacterial suspension was prepared in sterile saline at a concentration equivalent to 0.5 McFarland, and the optical density was measured using a turbidimeter (MicroScanturbidetMiller, Siemens, USA) at a wavelength of 620 nm indicating an absorbance degree between 0.8 and 0.10 and at a concentration equivalent to 1.5–2.0 × 10^8^ CFU/ml. Subsequently, the bacterial suspension was inoculated into each root canal until it was filled using a 1 ml tuberculin syringe and NaviTip 30 G needle (Ultradent, South Jordan, UT, USA). The bacterial suspension was inoculated until an overflow of the bacterial suspension was observed in the apical portion. The root apex was immediately dried with sterile gauze to seal the foramina of each root with a posterior Estelite photoactivated resin (Tokuyama Dental Corporation, Tokyo, Japan), preventing bacterial leakage through the apex during inoculation. Finally, the teeth were incubated for 21 days for biofilm formation under microaerophilic conditions at 37°C.

### 2.5. Application of Photodynamic Therapy

For group 1, a solution of Chimiolux (0.005% methylene blue) (DMC, São Paulo, Brazil) was used as the photosensitizer. Prior to its application, the excess culture medium was removed from the root canals, for which it was irrigated with 2 ml of physiological solution (NaCl 0.9%) and dried with sterile paper cones. Next, 0.005% methylene blue photosensitizer was applied to each root canal with a tuberculin syringe and NaviTip 30 G needle (Ultradent, South Jordan, UT, USA) and left there for 5 min (preirradiation).

For group 2, a 0.005% malachite green solution was used as the photosensitizer. This solution was prepared by dissolving malachite green powder (malachite green oxalate) (HiMedia Laboratories Pvt. Ltd., Mumbai, India) in sterile distilled water to achieve that concentration and then filtered through a sterile membrane (pore diameter of 0.22 *μ*m; MS®, Tokyo, Japan) into a presterilized container for later use. Next, 0.005% malachite green photosensitizer was applied to each root canal with a tuberculin syringe and NaviTip 30 G needle without overflow and left there for 5 min (preirradiation).

After the preirradiation time of the photosensitizers, laser light irradiation was continued, for which the Therapy XT laser device (DMC, São Paulo, Brazil) with a wavelength of 660 nm (red) and a tip of 200 *μ*m in diameter (DMC, São Paulo, Brazil) was used.

For the control group, no treatment was performed, and the root canals were inoculated with *E. faecalis* ATCC 29212.

### 2.6. Microbiological Analysis

For *E. faecalis* seeding, the loop depletion method was used: first, 1 ml of physiological solution (NaCl 0.9%) was added to each cryovial tube, which in turn contained a paper cone and shaken for 60 seconds to dilute and homogenise the sample. Next, a representative sample of 10 *μ*l was taken with a sterile loop and seeded on Enterococcosel agar at 37°C for 48 h. After the incubation period, colony-forming units (CFU/ml) were counted for each group using the plate count method.

### 2.7. Statistical Analysis

The means and standard deviations of the continuous variables (CFU/ml) were calculated. The normality of data distribution was determined through histogram plots and the Shapiro–Wilk test. Inferential analysis between groups was performed using the one-way ANOVA. Finally, simple regression analysis was performed. All statistical tests were performed at a 95% confidence level (*p* < 0.05). The data were processed and analyzed using Stata v.15.

## 3. Results

### 3.1. Comparison of the Antibacterial Effect of the Red Laser

Group 1 had the lowest mean antibacterial contamination with 530 ± 581.3 CFU/ml, whereas group 2 had the highest contamination with 1990 ± 542.5 CFU/ml. The continuous data in all the groups were normally distributed, with *p* > 0.05. The one-way ANOVA showed that there were statistically significant differences between the RL + MB and RL + MG groups (*p* < 0.001) (Table 1).

### 3.2. Post Hoc Analysis of the Antibacterial Effect of the Red Laser

In the post hoc analysis, there were only significant differences in the antibacterial effect of group 1 vs. group 3 and group 2 vs. group 3, with *p* < 0.001 in both cases (Table 2).

### 3.3. Linear Regression of the Antimicrobial Effect of the Red Laser

**Figure 1:**
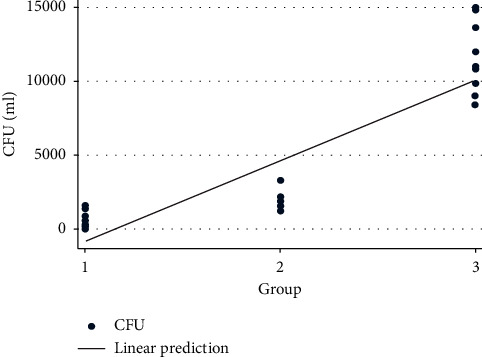
Linear regression analysis.

The average antibacterial efficacy was 1460 CFU/ml, CI (144.51–2775.4) in group 2, compared to group 1, with the difference being statistically significant. In addition, the average antibacterial efficacy was 10900 CFU/ml, CI (9584.51–12215.48) in the control group compared to group 1, with the difference being statistically significant (Figure 1 and Table 3).

## 4. Discussion

The mainstay of endodontics is complete disinfection of the root canal system. However, due to the resistance of the microorganisms present in root canals to conventional endodontic treatment, it has been demonstrated that their complete elimination is very difficult to achieve. *E. faecalis* is one of the most resistant microorganisms that cause secondary root canal infections. Therefore, photodynamic therapy has been suggested as an alternative and/or complement to conventional endodontic treatment because of its low toxicity mainly because the microorganisms do not present resistance to its therapeutic effects. Likewise, there are some variables to be considered when applying PDT, including the specific light source, photosensitizers, and irradiation parameters. For the microbiological analysis, the method used allowed to analyze the bacterial count through CFU/ml. However, since this was an in vitro study, the methodological design could be improved to be able to evaluate the variable microbial penetration into the root canals through electron microscopy images. [13–15].

The photosensitizers used in the present study were methylene blue and malachite green at a concentration of 0.005% for direct comparison. Both were coupled to a laser with a wavelength of 660 nm (red) for compatibility with the absorption spectrum. The laser device was used with a power of 100 mW for 90 s to obtain 9 J of energy. The control group was also evaluated. In the present study, the colony-forming unit count (CFU/ml), which is one of the most used methods for this type of study, was used to evaluate the antibacterial effect of the proposed treatments.

Our study aimed to evaluate the antibacterial effect of red laser using different photosensitizers on monoradicular human premolars contaminated with *Enterococcus faecalis* ATCC 29212. We found that the group 1 presented the lowest average antibacterial contamination while group 2 had the highest contamination, with the differences in concentration between the groups being statistically significant between groups. However, Sebrão et al. conducted a study comparing the efficacy of pink bengal and methylene blue photosensitizers in reducing the viability of *E. faecalis*, in which they used different concentrations and light sources: for methylene blue, they used a concentration of 31.2 *μ*mol/L (0.01%) associated with a laser of wavelength 660 nm (red) and for rose bengal, they used a concentration of 25 *μ*mol/L associated with a laser of wavelength 532 nm (green). Likewise, both laser devices were used with a power of 40 mW and for 180 s to obtain 7.2 J of energy. The methylene blue photosensitizer did not present significant differences with respect to the control, which differs from the results of the present study, where methylene blue differed significantly from the control [16].

Therefore, a factor to be considered in the study carried out by Sebrão et al. is that a lower amount of energy (7.2 J) was used with the laser device, which resulted in a lower bacterial reduction as opposed to the present study where the energy used was higher (9 J). Likewise, when comparing both photosensitizers with each other, they showed a significant difference, with rose bengal showing the best results.

On the other hand, Silva et al. [17] conducted a study comparing the antibacterial effect of methylene blue and malachite green, both with a concentration of 0.1% and associated with a laser with a wavelength of 660 nm (red). Likewise, the laser device was used with a power of 40 mW for 30, 60, and 120 s. It was found that both photosensitizers presented significant differences with respect to the control (60 and 120 s), which is similar to the results of the present study. However, when both photosensitizers were compared with each other, they showed no significant difference, which deviates from the results of the present investigation where methylene blue and malachite green, when compared with each other, showed statistically significant differences. In contrast to our study, Silva et al. used a higher concentration of the photosensitizers used (0.1%), which resulted in greater bacterial reduction as opposed to the present study where the concentration used was lower (0.005%). However, this concentration of 0.1% can stain the teeth, making it unsuitable for clinical application [18].

The main limitation of this in vitro study was that only two control groups were considered, although it would be important to compare with other disinfection methods. Another limitation to consider is that in that study [17], they did not use dental pieces, which may be clinically impractical, since, in the present study, dental pieces were used precisely to provide conditions as similar as possible to clinical conditions for both bacterial growth and the application of the treatments. Therefore, since the association of the red laser with both photosensitizers at a concentration of 0.005% (either methylene blue or malachite green) showed a significant antibacterial effect against *E. faecalis*, the possibility remains open for further studies of malachite green photosensitizers with new concentrations and specific light sources to promote their use as an alternative to the methylene blue photosensitizer in photodynamic therapy and its application in endodontics [19–21].

The present work seeks to increase evidence, as is the case with natural products [22, 23], on the antibacterial effect of the red laser associated with different photosensitizers such as methylene blue and malachite green against *E. faecalis* ATCC 29212, since the association between the two has shown good results against resistant microorganisms. Studies have been carried out on the application of red wavelength lasers associated with methylene blue due to the compatibility that exists between the two. However, very little has been studied on malachite green, even though this photosensitizer not only presents affinity with red wavelengths but can also be activated by other wavelengths such as blue and green, which gives it an advantage over other photosensitizers in terms of its application.

## 5. Conclusion

Within the limitations of the present in vitro study, it was found that the RL + MB group had the best antimicrobial potential because it presented the lowest contamination in CFU/ml of *E. faecalis* compared to the RL + MG group and the control group (no treatment).

## Figures and Tables

**Table 1:** Comparison of the antibacterial effect of the red laser on premolars contaminated with *E. faecalis* using different photosensitizers.

	CFU/ml					
	Mean	SD	Min	Max	*p* ^*∗*^	*p* ^∗∗^
RL + MB	530	581.3	0	1600	0.043	0.001
RL + MG	1990	542.5	1200	3300	0.086	
Control	11430	2352.3	8400	15000	0.371	

RL + MB: red laser associated with methylene blue photosensitizer; RL + MG: red laser associated with malachite green photosensitizer. ^*∗*^Shapiro–Wilk test; ^∗∗^ANOVA test.

**Table 2:** Post hoc analysis of the antibacterial effect of the red laser using different photosensitizers.

Groups	RL + MB	RL + MG
RL + MG	0.093	—
Control	0.001^*∗*^	0.001^*∗*^

^*∗*^Statistically significant.

**Table 3:** Linear regression of the antimicrobial effect of the red laser using different photosensitizers.

CFU/ml	Coef.	Std. err.	*p* value	95% conf. interval
RL + MB	Ref.			
RL + MG	1460	641.1275	0.031	144.5–2775.4
Control	10900	641.1275	0.001	9584.5–12215.4
Cons	530	453.3456	0.253	−400.1–1460.1

RL + MB: red laser associated with methylene blue photosensitizer; RL + MG: red laser associated with malachite green photosensitizer.

## Data Availability

The data that support the findings of this study are available from the corresponding author upon reasonable request.
